# Highlights from the Functional Single Nucleotide Polymorphisms Associated with Human Muscle Size and Strength or FAMuSS Study

**DOI:** 10.1155/2013/643575

**Published:** 2013-12-23

**Authors:** Linda S. Pescatello, Joseph M. Devaney, Monica J. Hubal, Paul D. Thompson, Eric P. Hoffman

**Affiliations:** ^1^Department of Kinesiology and Human Performance Laboratory, Neag School of Education, University of Connecticut, Gampel Pavilion Room 206, 2095 Hillside Road, U-1110, Storrs, CT 06269-1110, USA; ^2^Children's National Medical Center, 111 Michigan Avenue, N.W., Washington, DC 20010-2970, USA; ^3^Division of Cardiology, Hartford Hospital, 85 Jefferson Street, Hartford, CT 06106, USA

## Abstract

The purpose of the *Functional Single Nucleotide Polymorphisms Associated with Human Muscle Size and Strength* study or FAMuSS was to identify genetic factors that dictated the response of health-related fitness phenotypes to resistance exercise training (RT). The phenotypes examined were baseline muscle strength and muscle, fat, and bone volume and their response to RT. FAMuSS participants were 1300 young (24 years), healthy men (42%) and women (58%) that were primarily of European-American descent. They were genotyped for ~500 polymorphisms and completed the Paffenbarger Physical Activity Questionnaire to assess energy expenditure and time spent in light, moderate, and vigorous intensity habitual physical activity and sitting. Subjects then performed a 12-week progressive, unilateral RT program of the nondominant arm with the dominant arm used as a comparison. Before and after RT, muscle strength was measured with the maximum voluntary contraction and one repetition maximum, while MRI measured muscle, fat, and bone volume. We will discuss the history of how FAMuSS originated, provide a brief overview of the FAMuSS methods, and summarize our major findings regarding genotype associations with muscle strength and size, body composition, cardiometabolic biomarkers, and physical activity.

## 1. Introduction

We are part of a multidisciplinary research team, the Exercise and Genetics Collaborative Research Group, that completed a large exercise genomics study entitled *Functional Single Nucleotide Polymorphisms (SNPs) Associated with Human Muscle Size and Strength *(FAMuSS NIH R01 NS40606-02) [1]. The primary aim of FAMuSS was to identify non-synonymous SNPs (i.e., SNPs leading to amino acid changes) that dictated baseline muscle size and strength, and the extent of the muscle size and strength response to resistance exercise training (RT). Other phenotypes examined were baseline fat and bone volume and the response of these phenotypes to RT, and baseline cardiometabolic biomarkers. We envisioned that FAMuSS findings would lead to a better understanding of physical health and well being as well as disease processes such as sarcopenia during aging, atrophy during weightlessness of space flight, sports performance, and the progression of neuromuscular disease.

To achieve our aims about 1300 young, healthy men (42%) and women (58%) (24 years, body mass index [BMI] 25 kg·m^−2^) primarily of European-American descent were recruited and genotyped for ~500 polymorphisms. Volunteers provided blood samples for determination of fasting baseline cardiometabolic biomarkers and genotyping. They completed the Paffenbarger Physical Activity Questionnaire [2] to assess energy expenditure and time spent in light, moderate, and vigorous intensity physical activity and sitting. Subjects then performed a progressive, unilateral RT program of the nondominant arm with the dominant arm used as a comparison. Before and after RT, muscle strength was measured with the maximum voluntary contraction (MVC) and one repetition maximum (1RM), while magnetic resonance imagining (MRI) measured muscle, fat, and bone volume. To date there are over 30 FAMuSS publications. The purpose of this review is to highlight the FAMuSS findings by discussing the history of how FAMuSS originated, providing a brief overview of the FAMuSS methods, and summarizing our major findings regarding genotype associations with baseline muscle strength and size and body composition and the response of these phenotypes to RT, baseline fasting cardiometabolic biomarkers, and habitual physical activity levels.

## 2. The History of How FAMuSS Originated

Thomas A. Edison said, “Great Ideas Originate in the Muscles”. The study of skeletal muscle is not a new idea but one that has often intrigued human curiosity. If we begin to understand the complexities behind basic skeletal muscle function (i.e., strength) then this knowledge would provide insight into understanding normal human body movement and the ability to stress that system via an intervention. The FAMuSS study was an attempt to understand the genetic causes behind the response of muscle to an external stimulus. The aims of the FAMuSS project were to utilize molecular biology to answer two simple questions: (1) can genetic variation explain differences in skeletal muscle size and strength; and (2) can genetic variation explain how skeletal muscle responds to RT? The purpose of FAMuSS was not an attempt to understand the mechanical reasons for skeletal muscle size or strength but to comprehend the biology that controls the muscular apparatus.

Skeletal muscle makes up 30% of the human body so that the genetics behind the strength/size of this organ deserves attention. The maximum strength capacity of skeletal muscle is manipulated by a multitude of factors including genetics that can act synergistically. However, the most influential stimulus in the response of muscle is RT, which effectively increases maximal isometric and dynamic muscle contraction strength. Additionally, muscle strength is a key determinant of an individual's functional capacity. Even with the critical importance of skeletal muscle in human health, little was known regarding the genetic factors influencing skeletal muscle size and strength and the response of this organ to environmental factors such as RT. Thus, a comprehensive study was needed to discover how genetics influence skeletal muscle size and strength among healthy individuals as the paradigm at that time was studying genetic variation and its effect on dysfunction and disease. Our approach was not to lessen the need for the study of SNPs and disease—but to add new information to this important body of knowledge.

The FAMuSS study was built on the early work of Thomis et al. [3] that showed the inheritance of arm strength and size before and after RT in 25 monozygotic and 16 dizygotic male twins. Muscle strength measured as 1RM showed a high degree of heritability (77% pre- and 81% post-RT). Similarly, handgrip strength among 257 male and 353 female twins between 59–70 years suggested that strength had a heritability of 65% and 30%, respectively [4]. Pérusse et al. [5] used a statistical procedure, path analysis, which allows the partition of transmissible variance into genetic and cultural components among 1630 nontwin, French-Canadians from 375 families and attributed 30% of the phenotypic variance in muscular strength in these families to genetic factors. These findings made the rationale for the FAMuSS study even stronger. The identification of genetic variants that play a role in the normal response of muscle to external stimuli such as RT would have impact on the sports world, but more importantly, would also provide insight into health and disease processes permitting the possibility of new therapeutics to treat neuromuscular disorders.

## 3. The FAMuSS Methods

### 3.1. Overview

FAMuSS methods have been described in detail [1] and will be briefly overviewed here. FAMuSS was conducted by the Exercise and Genetics Collaborative Research Group consisting of researchers and site coordinators from the University of Central Florida (TJ Angelopoulos), University of Massachusetts (PM Clarkson), West Virginia University (PM Gordon), Dublin City University (NM Moyna), University of Connecticut (LS Pescatello), Central Michigan University (P Visich), Florida Atlantic University (RF Zoeller), Yale University (TB Price), Hartford Hospital (PD Thompson and RL Seip), and the Children's National Medical Center (EP Hoffman, PI, and JM Devaney). The institutional review boards from the 10 institutions involved in FAMuSS approved the study protocol. Study volunteers were recruited to complete a 12-week progressive, unilateral RT program to improve the strength and size of elbow flexor and extensor muscles in the nondominant arm with the dominant arm used as a comparison. Muscle strength was measured as biceps MVC and 1RM and muscle size by MRI of the biceps cross-sectional area. We used MRI to also measured fat and bone volume. Prior to RT, investigators obtained a blood sample for determination of a fasting cardiometabolic profile and DNA extraction, and subjects completed the Paffenbarger Physical Activity Questionnaire to assess habitual physical activity [2].

### 3.2. Subjects

Subjects were excluded if they took corticosteroids, anabolic steroids, antihypertensive or antilipidemic medications, diuretics, Depo-Provera contraceptive injection, Clenbuterol, Rhinocort nasal inhaler, lithium, or nonsteroidal anti-inflammatory medications. They were also excluded if they took dietary supplements to enhance muscle strength and size or weight; had chronic medical conditions; had metal implants in the arms, eyes, head, brain, neck, or heart; consumed >2 alcoholic drinks per day; performed RT or other physical activity involving repetitive arm use within the past year; and/or were seeking to gain or lose weight or had a weight change >5 lb in the past 3 months. Furthermore, subjects were instructed not to alter their habitual physical activity, lifestyle, or dietary habits, or otherwise gain or lose weight during the study. Upon enrollment we measured body weight and height to calculate BMI. To ensure weight maintenance, body weight was measured throughout the study.

### 3.3. Physical Activity Assessment

Habitual physical activity phenotypes were obtained from the Paffenbarger Physical Activity Questionnaire [2]. The derived phenotypes were energy expended (kcal·wk^−1^) and time spent (hr·wk^−1^) in light, moderate, and vigorous intensity physical activity as well as walking, stair climbing, participation in sports and recreational activities, and sitting. A total physical activity index (kcal·wk^−1^) was also calculated.

### 3.4. Muscle Strength Testing

We assessed muscle strength with the MVC and 1 RM in the trained (nondominant) and untrained (dominant) elbow flexor muscles before and after RT. 

### 3.5. MRI Assessment of Muscle, Fat, and Bone Volume

MRI assessment of both upper arms (trained and untrained) was performed before and after RT. Fifteen 16 mm contiguous axial slices from each arm were taken from each arm independently. Scans for both arms were taken by Fast Spoiled Gradient Recalled and Fast Spin Echo with TE 1.8/TR 200 msec. We used Rapidia (INFINITT Inc, Seoul, Korea) for the volumetric analysis of the MRI images. Volume measures were taken using an anatomical landmark (metaphyseal-diaphyseal junction of the humerus) as our starting point and assayed the six 1 cm slices proximal to it.

### 3.6. Resistance Exercise Training Program

RT was performed unilaterally in the nondominant arm. Subjects attended supervised RT sessions twice weekly at least 48 hours apart for 12 weeks. The program consisted of five exercises designed primarily to train the elbow flexors and secondarily to train the elbow extensors for balance. At the start of RT, subjects performed three sets of 12 repetitions at 65–75% of 1RM. At week five sets were reduced to eight repetitions at 75–82% 1RM and at week 10 to six repetitions at 83–90% 1RM. Subjects took 2 seconds each for the concentric and eccentric phase of each repetition with a recovery between sets of 2 minutes.

### 3.7. Fasting Blood Sampling and Analyses

*Cardiometabolic Biomarkers*. Prior to RT, fasting blood samples were drawn and serum was separated by centrifugation at 1110 g for 10 min and frozen for further analysis of the cardiometabolic biomarkers by Quest Diagnostics. Cardiometabolic biomarkers included glucose, insulin, total cholesterol, low-density lipoprotein cholesterol [LDL], high-density lipoprotein cholesterol [HDL], and triglyceride levels. The homoeostasis model assessment (HOMA) was then calculated [6]. *DNA*. In addition, blood was drawn into vacutainer tubes containing ethylenediamine tetraacetic acid. These tubes were sent to Children's National Medical Center where DNA was extracted using Puregene kits (Gentra Systems, Inc., Minneapolis, MN). Genotyping in the FAMuSS study was completed using TaqMan allele discrimination assays that employed the 5′ nuclease activity of Taq polymerase. Both alleles were detected simultaneously using allele-specific oligonucleotides labeled with different fluorophores (VIC and FAM) and genotypes were determined automatically by the ratio of the two fluorophores used. For each SNP examined, a Taqman assay was used to genotype the 1300 samples. Therefore, we generated 650,000 genotypes for the 500 SNPs that were part of the FAMuSS study. Data were processed using SDS v2.3 software. All gels were called by two investigators, and if any disagreement in genotyping was found, the genotyping was repeated.

## 4. FAMuSS Findings: Muscle Strength and Size

**Figure 1:**
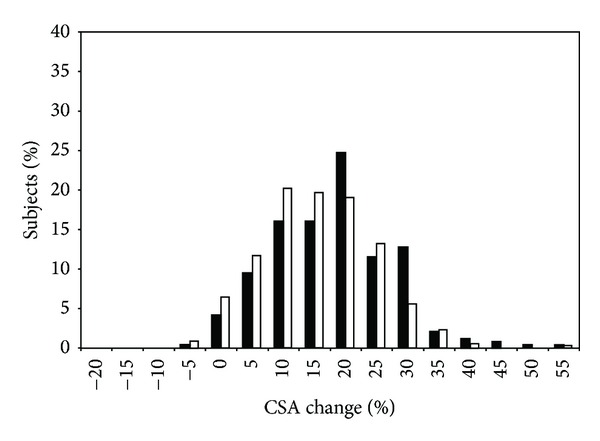
Biceps cross-sectional area.**   **Histogram of biceps cross-sectional area changes (relative to baseline) within each gender for the trained arm. Black bars denote responses of men, while white bars denote responses of women. Reprinted with permission [7].

**Figure 2:**
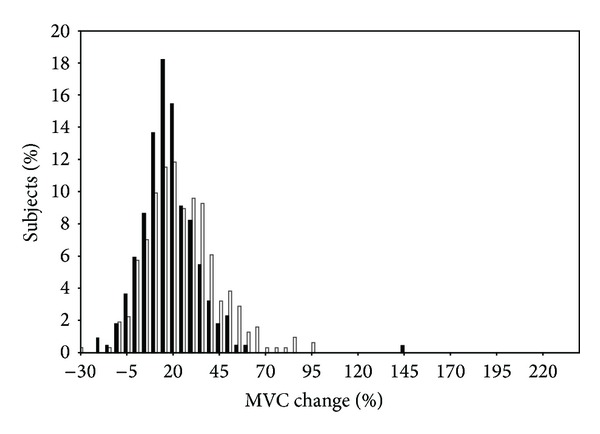
Isometric strength test.****Histogram of isometric strength changes (relative to baseline) within each gender for the trained arm. Black bars denote responses of men, while white bars denote responses of women. Reprinted with permission [7].

In 2005, Hubal et al. [7] published the results of the unilateral RT program on muscle size and strength in the FAMuSS cohort, highlighting the high degree of variability across all subjects given a standardized RT program. Across 485 subjects (342 women and 243 men), RT resulted in modest size gains and moderate (isometric) to high (dynamic) strength gains. Men averaged significantly higher absolute and relative size gains than women in the trained arm (Figure 1), while no significant changes were seen in the untrained arm. Size gains ranged from −2 to +59% of baseline muscle volume with similar distribution of responses in relative size gains between men and women. While absolute gains in strength (both dynamic and isometric strength) were greater in men, women greatly outpaced men for relative strength gains (64% to 40% for dynamic strength by 1 RM; 22% to 16% for MVC; *P* < 0.001) (Figure 2). Distribution parameters for strength gains indicated a strong clustering of men around the mean, while women ranged in a more normal distribution pattern, indicating that more women were higher or lower responders than men.

The high degree of variation that we observed in muscle size and strength responses to RT that were sex specific allowed us to test for various factors that influenced these phenotypes at baseline and in response to RT. While genetic modifiers of muscle strength and size were a priority for FAMuSS, other factors were also explored, such as sex [7], age [8], and BMI [9, 10]. However, the primary focus of this section is to summarize the findings regarding genetic influences on muscle size and strength at baseline and following RT. Twin and other genetic studies have reported wide-ranging estimates of heritability for baseline human muscle size (*h* ^2^ ~ 45–90%) and strength (*h* ^2^ ~ 30–85%) in a large part dependent upon the population and muscle group studied [11–13]. In addition to genetic influences on the development of muscle strength and size, there are other factors (i.e., training protocols, diet, etc.) that can modify the adaptive response of muscle to exercise training, such that even wider estimates of heritability are seen for hypertrophy and strength gains (*h* ^2^ ~ 35–85%) [11–13].

To date, the FAMuSS group has published results for 17 genes specifically tested for association with muscle strength or size that are summarized in Table 1 [14–26]. These genes can be categorized according to their biological functions, including muscle structural elements, growth factors, and inflammatory factors. Examples of structural genes include *ACTN3 *(actinin, alpha 3) [14] and *BMP2 *(bone morphogenetic protein 2) [15]; growth factors include *GDF8 *[growth differentiation factor 8 (myostatin, *MSTN*)] [20], *FST *(follistatin) [20], and *IGF1 *(insulin-like growth factor 1) [21]; and inflammatory factors include *CCL2 *[chemokine (C-C motif) ligand 2] [17], *IL15 *(Interleukin 15) [23], *IL15R*α** (interleukin 15 receptor, alpha) [23], and *SPP1 *(osteopontin or secreted phosphoprotein 1) [18], among others. A few genes from other biological function families (mainly related to blood flow and angiogenesis) have also been investigated that include *ACE* (angiotensin I converting enzyme) [19] and *NOS3 *(nitric oxide synthase 3) [16].

One structural gene variation that has garnered much attention is the common R577X (rs1815739) mutation in *ACTN3* [27], a premature stop codon that essentially eliminates ACTN3 protein expression in individuals with the XX (nonancestral) genotype. The ACTN3 protein is a sarcomeric actin-anchor expressed exclusively in Type II muscle fibers. The loss of this protein has been associated with athletic performance, such that the frequency of the mutation is underrepresented in elite power athletes and overrepresented in endurance athletes [28]. While animal knockout models have suggested compensatory upregulation of the similar alpha actinin 2 (*Actn2*) gene and possible alterations in aerobic energy pathway elements  [29, 30], these are yet to be confirmed in human studies.

A myriad of studies have examined the effect of *ACTN3* R577X genotype on athletic parameters, with widely varying results. Many of these studies suffer from having inadequate sample sizes and are often done in subjects that have varied exercise-training experiences, which can greatly overshadow subtle genotype effects. We reported significant sex-specific findings for muscle strength, but not size, among 602 subjects (247 men; 355 women) from FAMuSS [14]. Women with the XX genotype had lower baseline MVC but greater increases in dynamic strength as compared to women with the RX genotype. In addition, among women,* ACTN3 *accounted for 2.2% of variability in baseline MVC and 1.8% of the variability in 1RM gain.

The FAMuSS group has reported genetic associations in several growth related genes in relation to both baseline and posttraining muscle traits [15, 20, 21]. Skeletal muscle growth and protein synthesis are controlled by several key signaling pathways, such as the phosphatidylinositol-3-kinase (PI3 K)/protein kinase B (AKT)/mammalian target of rapamycin (mTOR) pathway. IGF1 positively controls this pathway via initiation of signaling at the cell surface, affecting protein synthesis rates. Conversely, *MSTN* and its related genes are negative regulators of muscle growth via inhibition of the PI3 K/AKT/mTOR signaling pathway. Animal models in which *MSTN* expression is greatly reduced exhibit gross muscle hypertrophy [31].

In the FAMuSS cohort, Kostek et al. [21] found associations of one particular promoter mutation in *IGF1* (rs35767) with muscle measures. Caucasian women with the CT genotype had greater baseline dynamic strength compared to the two other genotype groups.

In another study, Kostek et al. [20] found ethnicity-specific associations between *MSTN* (rs1805086) and muscle traits. A small group of African Americans showed greater baseline MVC in those with the G allele (*N* = 15) as compared to subjects with the AA genotype (*N* = 8), while no associations were found among Caucasians (*N* = 645). They also found associations between *FST* (rs722910) and strength measures among African Americans but not Caucasians. Finally, Devaney et al. [15] described associations between muscle size and a common polymorphism in *BMP2* (rs15705), which is known to inhibit myogenic differentiation [32]. Following RT, subjects with the CC genotype (*N* = 10) had significantly greater muscle volume gains compared to A allele carriers (*N* = 179), with 3.9% of trait variation explained by *BMP2*. Devaney et al. [15] noted that reporter assays specific for each allele showed that the C allele disrupted a posttranslational regulatory motif, possibly resulting in reduced inhibition, thereby allowing more muscle growth.

*BMP2* can be considered both a growth-related gene and an inflammation-related gene, based on its role in the transforming growth factor beta (TGFB) pathway. Inflammation and growth pathways have substantial overlap, fitting with the idea that postdevelopmental growth is in large part modulated by environmental stimuli such as exercise. Exercise that evokes the inflammatory system, such as loading muscles with lengthening (eccentric) muscle actions, often produces the greatest size gains [33].

Other key inflammatory genes investigated in FAMuSS for associations with muscle strength and size include *IL-15*, *IL15R*α*,* and *SPP1*. Pistilli et al. [23] reported associations between *IL15R*α** (rs2228059) and baseline muscle size in men but not women. This report also showed various relationships among *IL15* or *IL15R*α** and strength gains, including *IL15* (rs1057972) with strength gains in men and *IL15R*α** (rs2296135) with strength gains in women. Most recently, Hoffman et al. [34] reported a stronger association than is normally observed for exercise genomic studies between *SPPI* (rs28357094) and muscle size gains in women but not men [35]. The G allele was associated with increased size gains in women following RT, explaining a relatively high 5% of variance in the response.

In conclusion, despite the relatively strong association Hoffman et al. [34] observed with muscle size gains in women with *SPP1* after RT, in general, single variants explained minor trait variability percentages in baseline muscle size and strength and the response of these phenotypes to RT in the FAMuSS study [35]. Although it is possible that interactions between multiple genetic loci could have accounted for more trait variability [36, 37]. These genotype-phenotype associations were also often sex specific. From a clinical standpoint, genetic modifiers of muscle size and strength are already being studied in relation to management of various myopathies. For example, *SPP1* is a known modifier of disease severity in Duchenne muscular dystrophy [18]. Further studies into genetic influences on muscle size and strength (and their response to exercise training) will inform treatment options given an individual's genetic background, an example of personalized medicine. While these studies often involve muscle at pathological ends of the muscle size and strength spectra, FAMuSS findings provide a very valuable window into variant influences in “normal” (i.e., nonpathological) subjects. In addition, athletes will also seek advantage over their opponents using genomic medicine techniques such as exon-skipping to restore dystrophin expression in Duchenne muscular dystrophy that increases “natural” muscle size and strength possibly improving performance [38].

## 5. FAMuSS Findings: Body Composition and Cardiometabolic Biomarkers

As part of the FAMuSS study, body composition measurements were made such as BMI and MRI-dictated volumes of subcutaneous fat and bone of the upper arms before and after RT. In addition, before RT, measures of fasting glucose, insulin, total cholesterol, LDL, HDL, and triglyceride levels were made, and the HOMA was calculated [6]. To date, the FAMuSS group has published results for 33 genes specifically tested for association with measures of body composition at baseline and in response to RT and baseline cardiometabolic biomarkers that are summarized in Table 2 [22, 23, 39–43]. Some of these genes were also examined for skeletal muscle phenotypes pre- and post-RT that were described in the previous section and habitual physical activity that will be described in the next section so that only the findings relating to body composition and cardiometabolic biomarkers will be discussed in this section.

The metabolic syndrome is considered to be a pre-diabetic state, with elevated values for three out of five of the following cardiometabolic risk factors: blood pressure, waist circumference, blood glucose, triglycerides, and HDL [44]. The metabolic syndrome predisposes people to diabetes mellitus and cardiovascular disease. The FAMuSS study collected these cardiometabolic biomarkers among young, healthy adults, an optimal time for the implementation of lifestyle interventions to prevent disease progression [44, 45] as well as avoid the confounding effects of aging and its associated comorbidities on heritability [35].

Pistilli et al. [22] examined the influence of *RETN* (Resistin), a gene that has a potential role in inflammatory processes and metabolic diseases such as obesity, diabetes mellitus, and cardiovascular disease [46] on measures of body composition at baseline and in response to RT. *RETN* variants (rs34124816, rs1862513, rs3219177, rs3745367, and rs3219178) were strongly associated with muscle strength and muscle, bone, and fat volume phenotypes in men and women, but only when stratified by a BMI ≥ 25 kg·m^−2^, and they explained a relatively strong proportion of the variance in these phenotypes ranging from 7% to 12%. This study is evidence of the complex interactions that can exist among genes and measures of body composition [35].

BMP2 (bone morphogenetic protein 2) regulates the differentiation of pluripotent mesenchymal cells and inhibits myogenesis. In addition, high BMP2 levels promote osteogenesis or chondrogenesis and low levels promote adipogenesis. The interrelationships of muscle, fat, and bone cell deposition are key factors in both normal morphologic variation and a variety of medical conditions including the metabolic syndrome, vascular calcification, and osteoporosis [47, 48]. Devaney et al. [15] discovered sex-specific associations with *BMP2* (rs15705) and baseline subcutaneous fat volume and the response of subcutaneous fat and bone volume to RT. In addition, *BMP2* explained 2–4% of the variability in these phenotypes.

Due to rapid advances in field of genomics, new genetic tools became available while FAMuSS was being conducted. This work stemmed from two important achievements: (1) the completion of the Human Genome Project and (2) provision of an initial catalogue of human genetic variation and a haplotype map (HapMap, http://hapmap.ncbi.nlm.nih.gov/) [49]. These two important achievements coupled with the rapid improvements in genotyping technology and analysis led to genome wide association studies (GWAS). The FAMuSS study sought to leverage GWAS studies for variants that were identified to be associated with BMI and type 2 diabetes mellitus. We utilized the FAMuSS study to determine if these GWAS-identified variants would be associated with baseline adiposity, bone, and skeletal muscle phenotypes and the response of these phenotypes to RT as measured by MRI. In addition, we asked the question “do GWAS variants associated with BMI, lipid phenotypes, type 2 diabetes mellitus, and other cardiometabolic risk factors and diseases affect how an individual responds to exercise?”.

Orkunoglu-Suer et al. [41] examined one of the first variants identified using GWAS that was associated with obesity as denoted by BMI, *INSIG2* (insulin-induced gene 2) (rs7566605), for association with baseline subcutaneous fat volume and the response of this phenotype to RT. They found sex-specific associations with *INSIG2* and subcutaneous fat volume at baseline and in response to RT that accounted for <1–2.3% of the variance in subcutaneous fat volume.

GWAS was utilized to identify eight SNPs associated with BMI that highlighted a possible neuronal influence on the development of obesity [50]. These variants were *FTO *(fat mass and obesity associated) (rs9939609), *GNPDA2 *(glucosamine-6-phosphate deaminase 2) (rs10938397), *KCTD15 *(potassium channel tetramerisation domain containing 15) (rs11084753), *MC4R *(melanocortin-4 receptor) (rs17782313), *MTCH2 *(mitochondrial carrier 2) (rs10838738), *NEGR1 *(neuronal growth regulator 1) (rs2815752), *TMEM18* (transmembrane protein 18) (rs6548238), and *SH2B1 *(SH2B adaptor protein 1) rs7498665). Orkunoglu-Ser et al. [42] found sex-specific associations with *MC4R *(rs17782313) and BMI; *TMEM18* (rs6548238) and baseline subcutaneous fat volume; and *FTO* (rs9939609) and *SH2B1* (rs7498665) and the response of subcutaneous fat volume to RT. Collectively, these variants explained <1-2% of the variance in these body composition phenotypes.

The first gene examined in FAMuSS for associations with cardiometabolic phenotypes was *PPAR*α** (peroxisome proliferator-activated receptor alpha) that is involved in adipocyte differentiation and lipid and lipoprotein metabolism [43]. Studies in mice have shown that PPAR*α*-deficient animals were unable to metabolize lipids and develop late onset obesity even when kept on a stable diet [51, 52]. Uthurralt et al. [43] examined one of the most studied *PPAR*α** variants, a missense SNP in exon five that results in the amino acid substitution, leucine 162 valine (L162V; rs1800206). Uthurralt et al. [43] found European-American men with the V allele had higher baseline triglyceride levels and arm subcutaneous fat volume and lower HDL and tended to increase arm subcutaneous fat volume following RT compared to men with the LL genotype. The strength of the association with triglycerides was noteworthy with the V allele accounting for 4% of the variance.

IL-15 has influence on muscle-to-adipose tissue pathways as well as lipid and glucose metabolism [53]. Pistilli et al. [23] examined associations among genetic variants in *IL-15* and its receptor *IL-15R*α** with baseline cardiometabolic biomarkers and skeletal muscle, subcutaneous fat, and bone phenotypes at baseline and in response to RT. Sex-specific associations were found with *IL-15* and baseline total cholesterol (rs1589241), LDL (rs1589241), HOMA (rs1589241), BMI (rs1057972), glucose (rs1057972), and triglycerides (rs2228059) levels. In addition, men showed associations with *IL-15* and *IL-15*α** and baseline total bone volume (rs2296135) and cortical bone volume (rs2228059) as well as measures of muscle volume (rs2228095) and strength (rs1057972).

Converging lines of evidence suggested that AKT1 (V-akt murine thymoma viral oncogene homolog 1) was a major mediator of the responses to insulin, IGF1, and glucose. In addition, AKT1 has a key role in the regulation of muscle cell hypertrophy and atrophy. Devaney et al. [39] sought to validate associations with *AKT1* and metabolic syndrome phenotypes found in FAMuSS within three other study populations [Strong Heart Study (SHS) (*n* = 2,134; 55.5 ± 7.9 years), Dynamics of Health, Aging and Body Composition (Health ABC) (*n* = 3,075; 73.6 ± 2.9 years), and Studies of a Targeted Risk Reduction Intervention through Defined Exercise (STRRIDE) (*n* = 175; 40–65 years)]. They found that a three-SNP (rs1130214, rs10141867, and rs33926946) *AKT1* haplotype (i.e., a specific combination of neighboring alleles that tend to be inherited together) associated with fasting glucose levels among women in FAMuSS and with other metabolic phenotypes among women and men in the other three study populations. This study was an early attempt by the FAMuSS study investigators to functionally validate genetic associations that were previously discovered, for the validation of phenotype-genotype associations is an important prerequisite to better understand disease risk and provide therapeutic interventions that are often lacking in the field of exercise genomics [36].

Devaney et al. [40] analyzed 20 GWAS-identified SNPs associated with cardiometabolic risk factors in younger populations that included FAMuSS and a cohort of 6th grade children (Cardiovascular Health Intervention Program; CHIP). They established that the 1p13.3 LDL locus (rs646776) (near *SORT1*,   sortilin 1) was associated with LDL in both of these young populations. The variance accounted for by *SORT1* was considerably higher in these young populations (2.5%–4.1%) compared to older subjects from GWAS studies (1%).

In summary, the FAMuSS study discovered and validated numerous loci for associations with measures of body composition and cardiometabolic biomarkers. The genetic variants we examined explained <1–12% of the variance in the phenotypes examined suggesting these traits are highly polygenic with many loci contributing to a very small proportion of the variation. Furthermore, many of the genotype-phenotype associations were sex specific. More recently, the FAMuSS study began to mine GWAS studies to find, explore, and in some instances validate the impact of identified loci on a younger population that represents a critical period for therapeutic intervention as well as minimize the confounding effects of age on these phenotypes. In this way, the variance accounted for by genotype was higher in FAMuSS than GWAS that typically involve older subjects.

## 6. FAMuSS Findings: Physical Activity

Examining genetic variants that associate with habitual physical activity termed *physical activity genetics* was not a primary purpose of FAMuSS. Nonetheless, FAMuSS presented us with the opportunity to contribute to a growing body of literature showing the effect-mediation genetic variants associated with physical activity may have on chronic diseases and health conditions such as type 2 diabetes mellitus [54, 55], hypertension [36], and in the case of FAMuSS, overweight and obesity [41, 42, 56–59].

Over 67% of Americans are overweight to obese [60]. Physical inactivity is a major contributor to overweight and obesity as 74% of Americans do not meet the physical activity recommendations for weight maintenance [61]. It is of interest that the control of voluntary movement resides in similar central neural pathways as energy intake, emphasizing the role of the central nervous system in the regulation of energy expenditure and intake, and ultimately weight control [50, 59]. The redundancies in the etiology and control of physical activity and obesity led us**  **to test the hypothesis that genetic variants associated with obesity will associate with physical activity phenotypes derived from the Paffenbarger Physical Activity Questionnaire among the FAMuSS cohort.

The 11 genes reported to be associated with obesity phenotypes [25, 42, 50, 56, 58, 62, 63] that were tested for specific association with physical activity in FAMuSS are summarized in Table 3. Our work has revealed genotype differences in physical activity energy expenditure that ranged from about 500 to 2000 kcal·wk^−1^ that were dependent upon BMI, sex, and intensity or the level of physical exertion. These genotype differences have public health importance, equating to a potential weight gain or loss of 7–29 lb·yr^−1^. Furthermore, genotype accounted for ~1–5% of the variance in physical activity phenotypes substantiating the polygenetic influence on physical activity, and the large amount of heritability that remains unaccounted for [37].

Understanding the interactions between genetic variants associated with obesity and physical activity will provide insight into the causes and treatments of overweight and obesity. Our vision is that this research may eventually have important implications for a personalized approach to the prescription of physical activity for the treatment of overweight and obesity. For example, prescribing physical activity to reduce weight or maintain weight loss will be more effective if clinicians are able to create a unique prescription that targets the type or amount of physical activity an individual prefers to engage in based upon their genetic makeup. The vision is that such a personalized exercise prescription based upon this genetic information would facilitate physical activity adoption and adherence for that person [16, 24, 25, 35, 41, 42]. Nonetheless, due to the significant challenges in identifying genes and their regulatory factors that may influence overweight and obesity and their interactions with physical activity, a personalized approach for the prescription of physical activity for the treatment of this major public health epidemic is not evident for the immediate future.

## 7. Take-Home Messages, Future Directions, and Conclusions 

The FAMuSS study was an attempt to understand the genetic causes behind the response of muscle to an external stimulus, RT. The aims of the FAMuSS project were to utilize molecular biology to answer two questions: (1) can genetic variation explain differences in skeletal muscle size and strength? and (2) can genetic variation explain how skeletal muscle responds to RT? To date, the FAMuSS group has published results for 17 genes tested for association with muscle strength or size that have been categorized according to their biological functions that include muscle structural elements, growth factors, and inflammatory factors (Table 1). In general, single variants explained minor trait variability in baseline muscle size and strength and the response of these phenotypes to RT, indicating a polygenetic influence on these complex phenotypes, and many of these muscle size and strength genotype associations were sex specific. Moreover, FAMuSS findings have provided a very valuable window into variant influences in “normal” (i.e., nonpathological) young, healthy subjects.

In addition to its primary purpose, the FAMuSS group has published results for 33 genes tested for association with measures of body composition and their response to RT and baseline cardiometabolic biomarkers (Table 2) as well as 11 obesity genes tested for association with habitual physical activity levels (Table 3). The genetic variants that emerged from these analyses explained <1–12% of the variance in the phenotype examined, once again suggesting these traits are highly polygenic with many loci contributing a very small proportion of the variation, and these phenotype-genotype associations were often sex specific. More recently, the FAMuSS study mined GWAS studies to find, explore, and in some instances validate the impact of GWAS-identified loci on body composition and cardiometabolic biomarkers among a young population. In this way, the variance accounted for by genotype was higher in FAMuSS than GWAS involving older subjects partially due to the confounding effects of age being less in younger populations.

One persistent effect modifier of FAMuSS findings has been sex differences in the various phenotypes examined at rest and in response to RT. Hubal et al. [7] provided a detailed analysis of the variance of muscle strength and size responses in men and women to the 12 wk unilateral RT program, noting similar distributions in size gains (though men gained slightly more muscle volume than women in the trained arm) (Figure 1), but more variability in strength gains in women (as well as greater relative strength gains) (Figure 2). This increased variance in women for strength gains could account for some of the sex differences in the various phenotypes found in FAMuSS, as more high or low responders in a population could denote greater genetic influences. It is also possible that the greater amounts of androgens in men could account for a larger percentage of variation in responses, lowering the variance left to be accounted for by genetic factors. Furthermore, the response of a phenotype is often a function of baseline values that also varied by sex in FAMuSS [65]. In any case, sex-specific findings in genetic association studies are rather common, stemming from the large effect that biological sex has on a wide variety of phenotypes [35].

Our vision when we began FAMuSS was that with the identification of genetic variants that play a role in the normal response of muscle to external stimuli such as RT we would be able to better inform the sports world to maximize athletic performance and, more importantly, provide insight into disease processes—permitting the possibility of new therapeutics to treat neuromuscular disorders and other diseases and health conditions via a personalized medicine approach. What we have come to realize is that the journey to establish a personalized medicine approach to the treatment of disease that may also include a personalized approach to exercise prescription as lifestyle therapy was far more complex than anyone envisioned in 2001. Knowing what we have learned from the FAMuSS study and with the rapid advancement of technology since FAMuSS began in 2001, if we were to perform FAMuSS Part 2, we would (1) perform a GWAS and/or whole exome sequencing; (2) use an “interomic” approach to also measure the transcriptome, proteome, and metabolome at baseline and in response to RT to better capture gene expression; and (3) use bioinformatics combining quantitative with systems biology to conduct pathway analyses to elucidate mechanisms for the heritable factors and phenotype associations that emerge. Such an “interomic” bioinformatic approach would require a multidisciplinary team that has expertise in quantitative and systems biology as well as exercise physiology and preventive medicine. Nonetheless, FAMuSS has and will continue to have an important role in the journey to establish a personalized medicine approach to prevent, treat, and control disease as well as the development of new and more effective therapeutic options that will eventually be able to be prescribed on a more individualized basis.

## Figures and Tables

**Table 1:** FAMuSS findings: genetic loci associated with muscle size and strength at baseline and in response to resistance training.

Gene	Name	Reference
*ACE *	Angiotensin I converting enzyme	[19]
*ACTN3*	Actinin, alpha 3	[14]
*ANKRD6*	Ankyrin repeat domain 6	[24]
*BMP2*	Bone morphogenetic protein 2	[15]
*CCL2*	Chemokine (C-C motif) ligand 2	[17]
*CCR2*	Chemokine (C-C motif) receptor 2	[17]
*CNTF *	Ciliary neurotrophic factor	[26]
*FST *	Follistatin	[20]
*GDF8/MSTN *	Growth differentiation factor 8/myostatin	[20]
*IGF1*	Insulin-like growth factor 1	[21]
*IL15*	Interleukin 15	[23]
*IL* *15R*α**	Interleukin 15 receptor, alpha	[23]
*LEP *	Leptin	[25]
*LEPR *	Leptin receptor	[25]
*NOS3*	Nitric oxide synthase 3	[16]
*RETN *	Resistin	[22]
*SPP1*	Osteopontin or secreted phosphoprotein 1	[18]

**Table 2:** FAMuSS findings: genetic loci tested for association with body composition at baseline and in response to resistance training and cardiometabolic biomarkers at baseline*.

Gene	Name	Reference
***AKT1***	V-akt murine thymoma viral oncogene homolog 1	[39]
*ANGPT3 *	Angiopoietin-like 3	[40]
*BCL7B *	B-cell CLL/lymphoma 7B	[40]
***BMP2***	Bone morphogenetic protein 2	[40]
*CDKAL1 *	CDK5 regulatory subunit associated protein 1-like 1	[40]
*CDKN2A/2B *	Cyclin-dependent kinase inhibitor 2A and 2B	[40]
*CDKN2BAS/CDNKN2B-AS1* (*ANRIL*)	CDKN2B-AS1 CDKN2B antisense RNA 1	[40]
*CILP2 *	Cartilage intermediate layer protein 2	[40]
***FTO***	Fat mass and obesity associated	[42]
*GALNT2 *	UDP-N-acetyl-alpha-D-galactosame:polypeptide N-acetylgalactosaminyltransferase 2	[40]
*GNPDA2 *	Glucosamine-6-phosphate deaminase 2	[42]
*HHEX *	Hematopoietically expressed homeobox	[40]
*HNF1A *	HNF1 homeobox A	[40]
*IGFBP2 *	Insulin-like growth factor binding protein 2	[40]
***IL15***	Interleukin 15	[23]
***IL15R*α****	Interleukin 15 receptor, alpha	[23]
***INSIG2***	Insulin-induced gene 2	[41]
*KCTD10 *	Potassium channel tetramerisation domain containing 10	[40]
*KCTD15 *	Potassium channel tetramerisation domain containing 15	[42]
*KCNJ11 *	Potassium inwardly rectifying channel, subfamily J, member 11	[40]
***MC4R***	Melanocortin-4 receptor	[42]
*MRAS *	Muscle RAS oncogene homolog	[40]
*MTCH2 *	Mitochondrial carrier 2	[42]
*NEGR1 *	Neuronal growth regulator 1	[42]
***PPAR*α****	Peroxisome proliferator-activated receptor alpha	[43]
*PPARG2 *	Peroxisome proliferator-activated receptor gama	[40]
***RETN***	Resistin	[22]
***SH2B1***	SH2B adaptor protein 1	[42]
*SLC30A8 *	Solute carrier family 30, member 8	[40]
***SORT1***	Sortilin 1	[40]
*TCF7L2 *	Transcription factor 7-like 2	[40]
***TMEM18***	Transmembrane protein 18	[42]
*TRIB1 *	Tribbles pseudokinase 1	[40]

*Bolded genes were significantly associated with the phenotypes of interest.

**Table 3:** FAMuSS findings: genetic loci associated with habitual physical activity.

Gene	Name	Reference
*ANKRD6 *	Ankyrin repeat domain 6	[24]
*FTO *	Fat mass and obesity associated	[42]
*GHRL *	Ghrelin	[64]
*KCTD15 *	Potassium channel tetramerisation domain containing 15	[42]
*LEP *	Leptin	[25]
*LEPR *	Leptin receptor	[25]
*MC4R *	Melanocortin-4 receptor	[42]
*NEGR1 *	Neuronal growth regulator 1	[42]
*NOS3 *	Nitric oxide synthase 3	[16]
*SH2B1 *	SH2B adaptor protein 1	[42]
*TMEM18 *	Transmembrane protein 18	[42]
